# Leucine‐Rich Glioma‐Inactivated 1 versus Contactin‐Associated Protein‐like 2 Antibody Neuropathic Pain: Clinical and Biological Comparisons

**DOI:** 10.1002/ana.26189

**Published:** 2021-08-30

**Authors:** Sudarshini Ramanathan, Mandy Tseng, Alexander J. Davies, Christopher E. Uy, Sofija Paneva, Victor C. Mgbachi, Sophia Michael, James A. Varley, Sophie Binks, Andreas C. Themistocleous, Janev Fehmi, Yaacov Anziska, Anushka Soni, Monika Hofer, Patrick Waters, Fabienne Brilot, Russell C. Dale, John Dawes, Simon Rinaldi, David L. Bennett, Sarosh R. Irani

**Affiliations:** ^1^ Oxford Autoimmune Neurology Group, Nuffield Department of Clinical Neurosciences, John Radcliffe Hospital University of Oxford Oxford UK; ^2^ Neuroimmunology and Brain Autoimmunity Groups, Kids Neuroscience Centre, Children's Hospital at Westmead; Brain and Mind Centre and Sydney Medical School, Faculty of Medicine and Health University of Sydney Sydney New South Wales Australia; ^3^ Department of Neurology Concord Hospital Sydney New South Wales Australia; ^4^ Neural Injury Group, Nuffield Department of Clinical Neurosciences, John Radcliffe Hospital University of Oxford Oxford UK; ^5^ Inflammatory Neuropathy Group, Nuffield Department of Clinical Neurosciences, John Radcliffe Hospital University of Oxford Oxford UK; ^6^ Division of Neurology, Department of Medicine University of British Columbia Vancouver British Columbia Canada; ^7^ Wellcome Centre for Integrative Neuroimaging, Nuffield Department of Clinical Neurosciences, John Radcliffe Hospital University of Oxford Oxford UK; ^8^ Department of Neuropathology Oxford University Hospital, National Health Service Foundation Trust Oxford UK; ^9^ School of Medical Sciences University of Sydney Sydney New South Wales Australia; ^10^ T. Y. Nelson Department of Paediatric Neurology Children's Hospital Westmead Sydney New South Wales Australia

## Abstract

Pain is a under‐recognized association of leucine‐rich glioma‐inactivated 1 (LGI1) and contactin‐associated protein‐like 2 (CASPR2) antibodies. Of 147 patients with these autoantibodies, pain was experienced by 17 of 33 (52%) with CASPR2‐ versus 20 of 108 (19%) with LGI1 antibodies (*p* = 0.0005), and identified as neuropathic in 89% versus 58% of these, respectively. Typically, in both cohorts, normal nerve conduction studies and reduced intraepidermal nerve fiber densities were observed in the sampled patient subsets. In LGI1 antibody patients, pain responded to immunotherapy (*p* = 0.008), often rapidly, with greater residual patient‐rated impairment observed in CASPR2 antibody patients (*p* = 0.019). Serum CASPR2 antibodies, but not LGI1 antibodies, bound in vitro to unmyelinated human sensory neurons and rodent dorsal root ganglia, suggesting pathophysiological differences that may underlie our clinical observations. ANN NEUROL 2021;90:683–690

Patients with autoantibodies against leucine‐rich glioma‐inactivated 1 (LGI1) and contactin‐associated protein‐like 2 (CASPR2) most often present with autoimmune encephalitis^1^, ^2^, ^3^ and less commonly with peripheral nerve hyperexcitability (PNH) and/or dysautonomia^1^, ^4^, ^5^, ^6^ Pain has been described in approximately 10 to 30% of these patients^5^, ^7^, ^8^; however, prior studies have not utilized validated pain scores, quantified outcome measures, or investigated the underlying neurobiology. Herein, inspired by an index patient with LGI1 antibodies and immunotherapy‐responsive neuropathic pain, we systematically characterized clinical features, therapeutic responses, outcomes, and potential pathophysiological mechanisms in 147 patients with LGI1 and/or CASPR2 antibodies.

## Patients and Methods

### Clinical Characterization

Patients with antibodies against LGI1 (n = 108), CASPR2 (n = 33), or both targets (n = 6) were identified from the Oxford Autoimmune Neurology Group's clinical assessments, including 37 patients from previous studies.^9^, ^10^ From 39 patients with pain, case notes (in all 39) and additional telephone interviews (23/39) retrospectively assessed clinical features, including patient‐rated treatment responses (no response/worsening vs any improvement) and 3 validated questionnaires:

1. The Douleur Neuropathique 4 (DN4) was used to define neuropathic pain at disease nadir by a score ≥ 3 (without physical examination).^11^
2. Patient‐Reported Outcome Measurement Information System Pain Interference (PROMIS‐PI; maximal score = 40) was used to quantify pain interference at nadir of pain, after immunotherapy, and at latest follow‐up (median = 5 years, range = 1–17).
3. Five‐level EuroQol 5‐dimension quality of life assessment (EQ‐5D) and EQ‐5D visual analogue scale were used to evaluate functional domains and self‐reported quality of life (QOL; 0 worst to 100 best health) at latest follow‐up.

### Laboratory‐Based Characterization

Intraepidermal nerve fiber density (IENFD) was determined from skin biopsies^12^ and human leukocyte antigen (HLA) genotyping from blood,^9^ both as previously described.

The first available serum sample was tested using (1) live cell‐based assays (CBAs) for LGI1 and CASPR2 antibodies, with samples defined as positive at serum endpoint dilutions ≧1:20 for LGI1 and ≧1:100 for CASPR2 antibodies^1^; (2) immunohistochemistry (IHC) using rodent hippocampal sections; (3) live cocultures of human induced pluripotent stem cell (iPSC)‐derived sensory neuronal cultures myelinated by rat Schwann cells^13^, ^14^; and (4) live murine dorsal root ganglion (DRG) cultures.^15^ Samples were compared to the following age‐/sex‐matched controls: healthy controls (n = 12); and patients with characteristic central nervous system (CNS) manifestations but without pain, who have antibodies against LGI1 (n = 6), CASPR2 (n = 8), or both antigens (n = 2).

GraphPad (San Diego, CA) Prism (v8.0) and Adobe Illustrator were used for statistical analyses/figures. Fisher exact test was used to compare binary discrete and Mann–Whitney test to compare continuous variables. Informed consent from patients and controls was obtained with approvals REC16/YH/0013 and 14/SC/0280. Animal work complied with UK Home Office license Ref.P1DBEBAB9.

## Results

### Index Patient

A 66‐year‐old previously well female presented with 18 months of pain, which commenced as a hot sensation in both feet. Subsequently, she developed shooting pains in her thighs, buttocks, arms, and torso, stereotyped episodes of burning pain described as “being tortured with stinging nettles”, and persistent severe hyperesthesia with a glove and stocking distribution also involving her torso. Symptoms were triggered by heat and movement, and severely restricted activities. Three neurologists observed normal nerve conduction studies and diagnosed her with fibromyalgia or psychogenic pain. A fourth found LGI1 antibodies (endpoint dilution of 1:640) and markedly reduced skin IENFD. High‐dose prednisone resulted in complete symptom resolution within 2 days, and a marked fall in PROMIS‐PI ratings, from 36/40 to 8/40. Nine months later, a patient‐initiated corticosteroid wean was associated with a relapse of pain, which partially responded to reinitiation of corticosteroids and plasma exchange.

This patient prompted us to examine pain across a large cohort of patients with LGI1 and/or CASPR2 antibodies.

### Autoantibodies and HLA Genotypes

**FIGURE 1:**
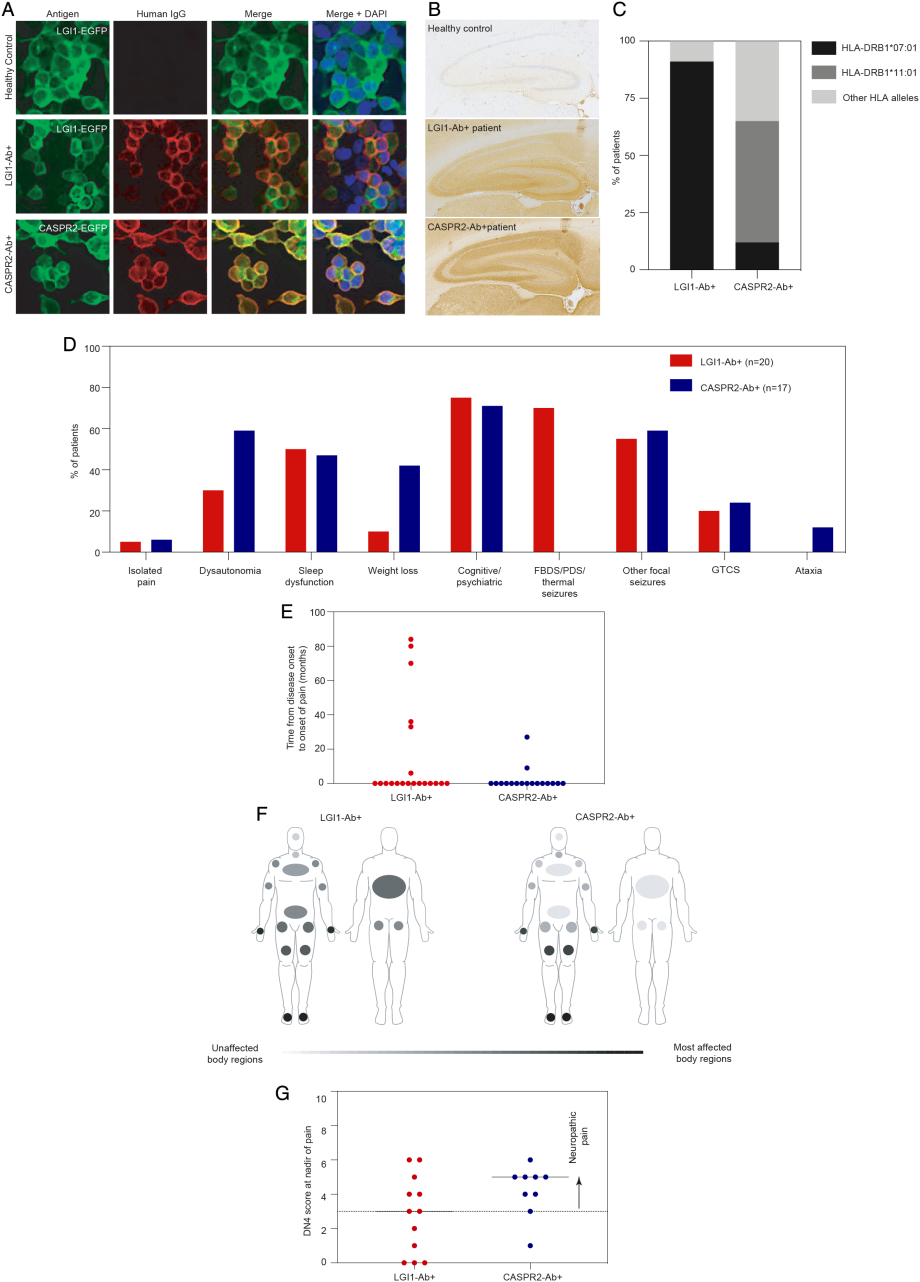
Clinical characterization of leucine‐rich glioma‐inactivated 1 antibody (LGI1‐Ab^+^) and contactin‐associated protein‐like 2 antibody (CASPR2‐Ab^+^) patients with pain. (A) Representative example of live cell‐based assay demonstrating HEK293T cells transfected with LGI1 or CASPR2 (green; tagged with enhanced green fluorescent protein [EGFP]) and colocalized human immunoglobulin G (IgG) binding (red) in patients with LGI1 antibodies, patients with CASPR2 antibodies, and a healthy control. (B) Representative example of sera from patients with LGI1 or CASPR2 antibodies, but not from healthy controls, binding to rodent hippocampal sections. (C) Ten of 11 (91%) LGI1 antibody patients with DNA available for testing had a known risk allele for human leukocyte antigen (HLA)‐DRB1*07:01 compared to 2 of 17 (12%) CASRP2 antibody patients (*p* < 0.0001). Nine of 17 (53%) CASPR2 antibody patients carried the known risk allele HLA‐DRB1*11:01 compared to 0 of 11 LGI1 antibody patients (*p* = 0.0039). (D) Features of 37 patients with LGI1 or CASPR2 antibodies and pain, including 2 with isolated pain, 2 with pain and dysautonomia/weight loss, and 33 with concomitant prominent central nervous system (CNS) manifestations. Faciobrachial dystonic seizures (FBDS), thermal seizure semiologies, and paroxysmal dizzy spells (PDS) were present exclusively in patients with LGI1 antibodies, whereas ataxia was only present in CASPR2 antibody patients. GTCS = generalized tonic–clonic seizures. (E) The majority of patients noted onset of pain contemporaneous with the onset of CNS manifestations. In 35% of patients with LGI1 antibodies and 12% with CASPR2‐antibodies, pain onset was delayed by a median interval of 33 months. (F) Twenty‐three of 37 patients completed pain questionnaires. The lightest gray shade represents regions with no pain, with increasingly dark shades of gray denoting more frequent pain localisation. Involvement of the trunk occurred exclusively in patients with LGI1 antibodies (42% vs CASPR2‐Ab^+^ 0%, *p* = 0.045), whereas limbs, face, and neck were affected at similar rates (limb, LGI1‐Ab^+^ 75% vs CASPR2‐Ab^+^ 78%; face/neck, LGI1‐Ab^+^ 17% vs CASPR2‐Ab^+^ 22%). (G) At disease nadir, both groups of patients had comparable Douleur Neuropathique 4 (DN4) scores (LGI1‐Ab^+^ with median = 3 and range = 0–6 vs CASPR2‐Ab^+^ with median = 5 and range = 1–6; *p* = 0.319). Fifty‐eight percent of LGI1 antibody–positive and 89% of CASPR2 antibody–positive patients had DN4 scores ≥ 3, consistent with neuropathic pain. Further data are presented in Table S1.

Antibodies against LGI1 (n = 108), CASPR2 (n = 33), or both targets (n = 6) were detected using live CBAs (Fig 1). Samples reaching endpoint dilutions of ≧1:80 for LGI1 antibodies and ≧1:800 for CASPR2 antibodies consistently showed reactivity by IHC. HLA genotypes were accordant with established associations for both disorders^9^: 10 of 11 (91%) LGI1 antibody patients carried HLA‐DRB1*07:01, and 9 of 17 (53%) CASPR2 antibody patients carried HLA‐DRB1*11:01.

### Clinical Features and Investigations

Thirty‐nine of 147 patients described pain as a component of their illness, including 52% (17/33) of the CASPR2‐antibody cohort versus 19% (20/108) with LGI1 antibodies (*p* = 0.0005), and 2 of 6 with both antibodies (Table S1). Isolated pain was observed in 2 patients (the index case with LGI1 antibodies, and another patient with CASPR2 antibodies); they carried HLA‐DRB1*07:01 and HLA‐DRB1*11:01, respectively, and their sera demonstrated high endpoint dilutions on live CBAs with characteristic binding on IHC. The remaining 37 of 39 (95%) described pain in addition to CNS features and/or systemic involvement (weight loss, dysautonomia; see Fig 1). Thirty of 39 patients developed pain concurrent with the onset of CNS symptoms. In 9 of 39 (23%; Fig 1E), pain began after onset of CNS features by a median of 33 months (range 2‐84). Fibromyalgia, psychogenic pain, or chronic fatigue syndrome were the original diagnoses in 7 of 39 (18%). Electrophysiologically confirmed PNH was present in 16 of 39 (41%) patients. Nerve conduction studies were normal in 29 of 39 (74%), with one quarter showing a mild sensory axonal polyneuropathy.

Questionnaires revealed that both the LGI1 and CASPR2 antibody cohorts had predominant length‐dependent distributions of pain, with truncal involvement exclusive to the LGI1 antibody cohort (5/12 [42%] vs 0/9 [0%] with CASPR2 antibodies; *p* = 0.045; Fig 1E). At nadir of pain, both groups had comparable DN4 and PROMIS‐PI scores with neuropathic pain in 7 of 12 (58%) LGI1 and 8 of 9 (89%) CASPR2 antibody patients (see Fig 1F and Table S1).

### Short‐ and Long‐Term Outcomes after Treatment

**FIGURE 2:**
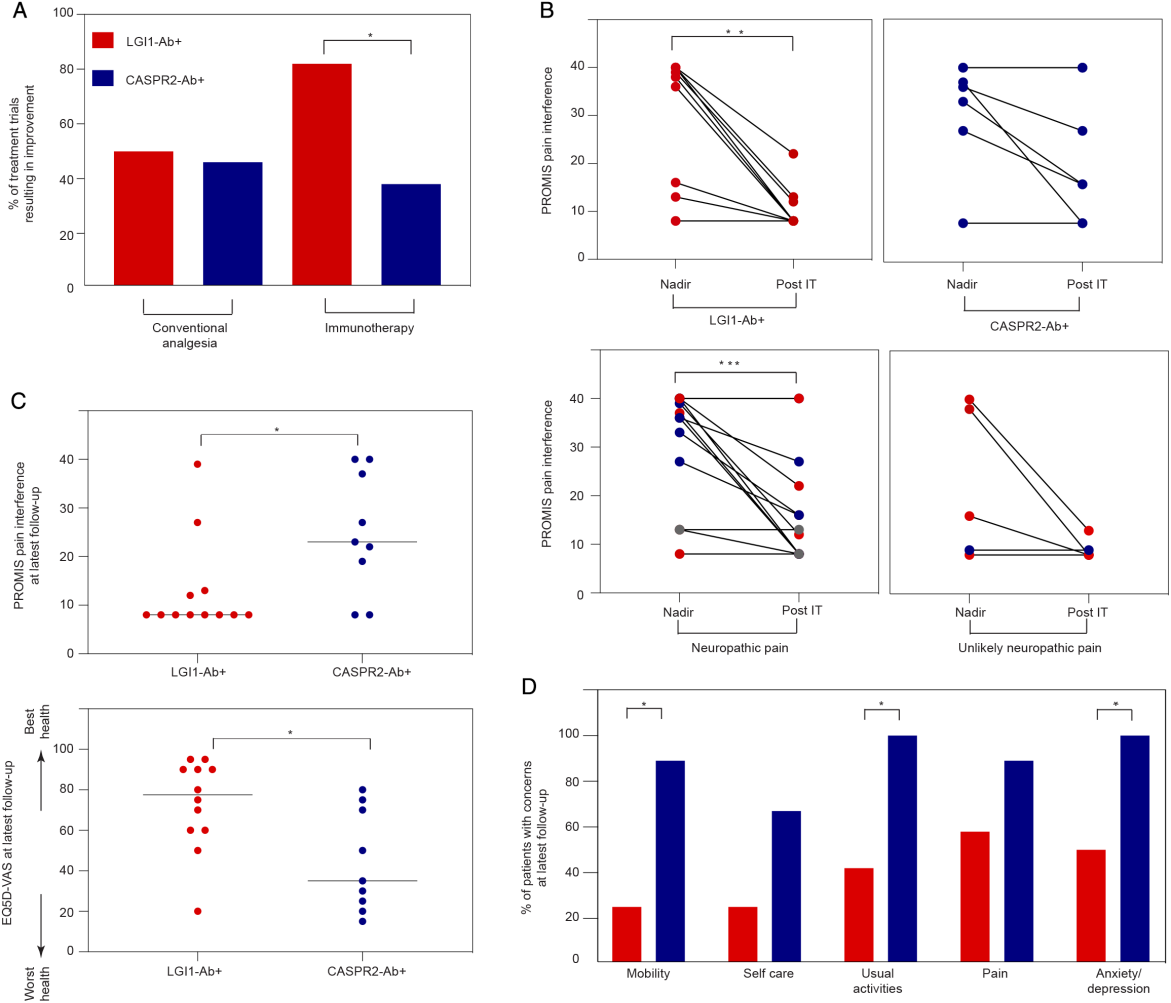
Therapeutic responses and functional outcomes of leucine‐rich glioma‐inactivated 1 (LGI1) and contactin‐associated protein‐like 2 (CASPR2) antibody patients with pain. (A) Patients with LGI1 antibodies more frequently reported improvements after immunotherapy than those with CASPR2 antibodies (LGI1‐Ab^+^ 82% vs CASPR2‐Ab^+^ 38%; *p* = 0.024). Responses in both groups were similar following conventional analgesia administration (LGI1‐Ab^+^ 50% vs CASPR2‐Ab^+^ 46%; *p* > 0.999). Conventional analgesic agents included, in descending frequency, pregabalin and gabapentin (n = 6 each) > amitriptyline (n = 4) > duloxetine/naproxen/buprenorphine/codeine (n = 2 each) > carbamazepine/sertraline/venlafaxine/phenytoin/lignocaine patches/paracetamol (n = 1 each). Immunotherapies included prednisone (n = 18), intravenous immunoglobulin (n = 7), plasmapheresis (n = 7), mycophenolate (n = 4), azathioprine/rituximab/omalizumab/thymectomy (n = 1 each). (B) Patient‐Reported Outcome Measurement Information System (PROMIS) pain interference ratings were similar between both antibody‐defined groups at nadir of pain (*p* = 0.662), but showed a more significant reduction following immunotherapy (IT) in those with LGI1 antibodies (*p* = 0.008) versus those with CASPR2 antibodies (*p* = 0.125). This effect was most pronounced for patients with neuropathic pain (*p* = 0.0005) compared to those with unlikely neuropathic pain (*p* = 0.25). Gray points identify two double‐positive patients. (C) At latest follow‐up (LGI1‐Ab^+^ median = 66 months, CASPR2‐Ab^+^ median = 57 months, *p* = 0.715), CASPR2 antibody patients had higher PROMIS pain interference ratings (LGI1‐Ab^+^ median = 8, range 8–39; CASPR2‐Ab^+^ median = 23, range 8–40; *p* = 0.025) and lower overall self‐reported health based on the EuroQol 5‐dimension quality of life assessment visual analogue scale (EQ‐5D‐VAS; LGI1‐Ab^+^ median = 78, range = 20–95; CASPR2‐Ab^+^ median = 35, range = 15–80; *p* = 0.019). Zero is worst and 100 is best health. (D) At follow‐up, CASPR2 antibody patients showed more impairment in 5‐level EQ‐5D ratings of mobility (*p* = 0.014), usual activities (*p* = 0.019), and anxiety/depression (*p* = 0.043). * p ≤ 0.05; ** p ≤ 0.01; *** p ≤ 0.001.

Patients were administered a median of 2 conventional analgesic agents (range = 1–6; most commonly pregabalin, gabapentin, and amitriptyline) and a median of 2 immunotherapies (range = 1–4, mostly prednisone and intravenous immunoglobulin or plasma exchange). Improvement of pain with conventional analgesic trials was reported in approximately half of both LGI1 (7/14; 50%) and CASPR2 antibody (6/13; 46%) patients. In contrast, 18 of 22 (82%) immunotherapy trials in LGI1 antibody patients resulted in a rapid improvement in pain, over a median of 14 days (range = 2–21), compared to 5 of 13 (38%) in CASPR2 antibody patients (*p* = 0.024; Fig 2, Table S1). Moreover, by comparison to the CASPR2 antibody cohort, the LGI1 antibody patients showed the most marked PROMIS‐PI reductions following immunotherapy (*p* = 0.008): this was particularly pronounced in those with neuropathic pain (*p* = 0.0005, Fig 2B). The lower PROMIS‐PI scores in LGI1 antibody patients were sustained at latest (median = 5 years) follow‐up (*p* = 0.025, Fig 2C) and, at this time point, CASPR2 antibody patients lower overall self‐reported health (*p* = 0.019), with greater concerns in the domains of mobility (*p* = 0.014), usual activities (*p* = 0.019), and anxiety/ depression (*p* = 0.043, Fig 2D).

### Intraepidermal Nerve Fiber Densities

**FIGURE 3:**
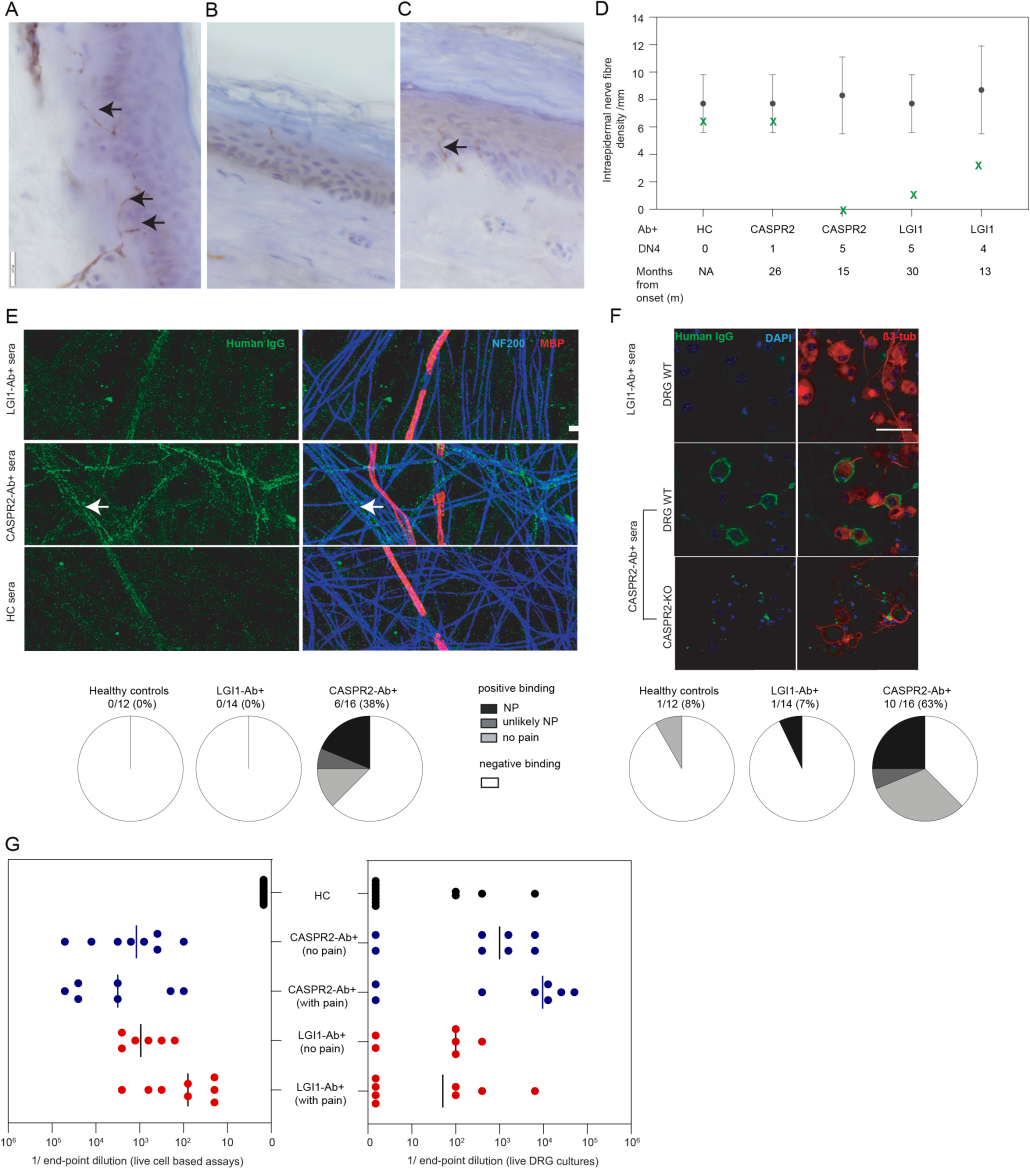
Intraepidermal nerve fiber densities and in vitro binding characterization of leucine‐rich glioma‐inactivated 1 (LGI1) and contactin‐associated protein‐like 2 (CASPR2) antibodies. (A) Healthy control skin biopsy showing nerve fibers crossing the dermoepidermal junction (*arrows*), identified with the pan‐neuronal marker protein gene product PGP9.5. Scale bar = 20μm. (B) A 70‐year‐old male with LGI1 antibodies and neuropathic pain with Douleur Neuropathique 4 (DN4) score = 5 had a markedly reduced median intraepidermal nerve fiber density (IENFD) of 0.87/mm. (C) The index case, a 67‐year‐old female with isolated neuropathic pain and LGI1 antibodies and DN4 score = 4, had a reduced median IENFD of 2.57/mm (*arrow*). (D) Summary of IENFD for each individual case (*green crosses*) and the healthy control age‐ and gender‐matched median IENFD (*black dots*) with 0.05 quantile ranges (*black lines*). (E) Serum immunoglobulin G (IgG) from 6 of 16 CASPR2 patients (with neuropathic pain [NP], unlikely NP, and no pain) bound to unmyelinated human sensory axons (blue, neurofilament heavy chain [NF200]; red, myelin basic protein [MBP]; and green, patient IgG) compared to 0 of 14 LGI1 patients with and without NP (*p* = 0.019) and 0 of 12 age‐/sex‐matched healthy controls (HC). White arrows demonstrate IgG binding to unmyelinated sensory neurons. Seventeen of 28 (61%) healthy controls plus seropositive cases without pain were male, median age = 63 years, range = 22–84. Serum samples were scored as showing positive or negative immunoreactivity to the cocultures by an observer blind to the patient group. Scale bar = 10μm. (F) Serum IgG from 10 of 16 CASPR2 antibody patients bound to wild‐type (WT) mouse dorsal root ganglion (DRG) cultures at endpoint dilutions > 1:400, compared to 1 of 14 LGI1 patients (*p* = 0.0024) and 1 of 12 healthy controls. Binding of human IgG colocalized with nucleated (blue, DAPI) rodent WT DRG neurons (red, β3‐tubulin), but not to DRG neurons derived from CASPR2 knockout mice (CASPR2‐KO). Scale bar = 50μm. (G) Endpoint dilutions tested on live cell‐based assays and on live DRGs for both LGI1 and CASPR2 antibody sera did not demonstrate significant differences between patients with and without pain.

Four patients underwent IENFD evaluation at a median of 20 months (range = 13–30) following onset of pain (Fig 3A–D). One patient with CASPR2 antibodies, CNS involvement, and non‐neuropathic pain (DN4 score = 1) had an IENFD in the healthy control range. This contrasted with strikingly low, sometimes absent, IENFDs in 3 patients with LGI1 or CASPR2 antibodies, all with neuropathic pain and DN4 scores of 4–5 (see Fig 3D).

### Binding to Live Neuronal Cultures

LGI1/CASPR2 antibody titers by live CBA, and their binding to extracellular domains expressed on neurons with the potential to mediate small fiber pain, were assessed from the 16 of 39 patients with pain and sufficient sera available, and from 26 controls. By contrast to LGI1 antibody and healthy control sera, CASPR2 antibody sera showed a higher frequency of binding to both unmyelinated axons of live iPSC‐derived human sensory neurons (LGI1 antibody 0/14 [0%] vs CASPR2 antibody 6/16 [38%], *p* = 0.019) and live rodent DRG neurons (LGI1 antibody 1/14 [7%] vs CASPR2 antibody 10/16 [63%], *p* = 0.0024). All CASPR2 antibody binding was abrogated in DRG cultures derived from a CASPR2 knockout mouse (see Fig 3E, F). The binding and endpoint dilutions of LGI1 and CASPR2 antibodies were similar between patients with and without pain (see Fig 3G).

## Discussion

This study shows neuropathic pain as a frequent and often rapidly immunotherapy‐responsive feature in a large cohort of patients with LGI1 and CASPR2 antibodies, including ~10% with relatively isolated pain syndromes. A comparison of patients with LGI1 versus CASPR2 antibodies revealed that length‐dependent pain was frequent in both groups, whereas truncal involvement was exclusive to LGI1 antibody patients. The LGI1 antibody patients demonstrated more dramatic responses to immunotherapy, often observed within a few days as highlighted by our index case. Their preferential response to immunotherapy over conventional symptomatic treatments is reminiscent of the superior effect of immunotherapy versus anticonvulsant therapy in LGI1 antibody–associated seizures.^10^ Importantly, this benefit was sustained at 5‐year follow‐up in LGI1 antibody patients. The higher rate of residual immunotherapy‐resistant pain in CASPR2 antibody patients interfered with activities of daily living and was associated with reduced QOL.

LGI1 and CASPR2 are expressed in the CNS and also in the peripheral nervous system, including peripheral nerves and DRGs.^15^, ^16^, ^17^ Our observations, and those of others,^5^, ^7^, ^8^, ^15^, ^18^ suggest small nerve fiber dysfunction may underlie our patients' pain, as supported by the distal limb predominance and neuropathic quality with typically both unremarkable nerve conduction studies and large nerve biopsies.^8^ Furthermore, we show IENFD can be markedly reduced in both LGI1 and CASPR2 antibody patients, similar to prior observations from CASPR2 antibody patients.^7^ A direct effect on peripheral sensory neurons is supported by the DRG hyperexcitability observed in rodents administered CASPR2 immunoglobulin Gs (IgGs) from 2 patients.^15^ Here, we extend these data by showing that serum IgGs from many CASPR2 antibody patients bind with high frequency to both rodent DRG neurons and, for the first time in human tissue, iPSC‐derived unmyelinated sensory nerve axons. The neuronal IgG binding from CASPR2 antibody patients without pain suggests additional factors may regulate their neuropathic potential in vivo, for example, blood nerve barrier access. Additionally, our data suggest CASPR2 IgGs are unable to access the juxtaparanode underneath the myelin sheath, as demonstrated by the undetectable IgG binding to our in vitro myelinated axons. Overall, the high rate of CASPR2 IgG binding may underly the more frequent and sustained pain observed in this cohort.

The absence of LGI1 antibody binding to both preparations may be because LGI1 is a soluble molecule and hence may not be retained in vitro.^16^ Alternatively, a CNS action of LGI1 antibodies may mediate pain consistent with observations including the frequent concurrent encephalopathy, striking binding to CNS tissue, and the truncal distribution reminiscent of central cord syndromes. The molecular basis for frequent immunotherapy‐mediated pain resolution despite small nerve fiber loss requires further study.

Our work methodologically advances the field with (1) the first structured neuropathic pain evaluations using validated clinical and laboratory tools; (2) ascertainment of transferable measures of QOL for future cross‐comparisons; (3) long‐term follow‐up data; and (4) the study of strictly autoantibody‐mediated conditions, by exclusion of voltage‐gated potassium channel antibody–positive patients without LGI1/CASPR2 reactivities.^8^, ^18^, ^19^ Limitations include the inherent caveats of a retrospective cohort review and the potential for recall bias in patients with residual cognitive impairment.

In summary, here, by contrasting LGI1 and CASPR2 antibody patients, we advance established differences between their clinical presentations and HLA associations to now differentiate their pain in terms of its relative frequency, clinical characteristics, short‐ and longer‐term therapeutic responses, and the underlying neurobiology. Our detailed characterization of "autoimmune pain" syndromes^20^ aims to ensure appropriately selected patients are directed toward early proactive immunotherapy to limit long‐term morbidity and associated disability, and suggests that autoantibody testing in cohorts with a prior diagnosis of fibromyalgia or psychogenic pain may identify a modest subset with immunotherapy‐responsive pain^20^ and inform their biology.

## Author Contributions

S.Ra. and S.R.I. contributed to conception and design of the study, as well as acquisition and analysis of data and drafting a significant portion of the manuscript or figures. All coauthors contributed to acquisition and analysis of data, in addition to editing and approval of the final draft.

## Potential Conflicts of Interest

J.D. and D.L.B. have a patent relating to the use of CASPR2 for the treatment of excess neuronal activity in pain and epilepsy (PCT/GB2017/052909). S.R.I. and P.W are coapplicants and receive royalties on a licensed patent application WO/2010/046716 (UK patent no. PCT/GB2009/051441) entitled "Neurological Autoimmune Disorders." All other authors have no relevant disclosures.

## Supporting information


**TABLE S1.** Comparison of leucine‐rich glioma‐inactivated 1 (LGI1) and contactin‐associated protein‐like 2 (CASPR2) antibody–positive patients with pain. ^a^Fisher exact test was used to compare binary discrete variables between the LGI1 and CASPR2 pain groups. The Mann–Whitney test was used to compare continuous variables. Probability values are not corrected for multiple comparisons. Double‐positive patients were excluded from statistical analysis due to the small sample size. ^b^Douleur Neuropathique 4 (DN4) scores out of 10 (2 points were discounted for lack of physical examination). Scores > 3 are considered to represent neuropathic pain in the absence of physical examination. ^c^Patients who had multiple trials of conventional analgesia or immunotherapy agents were classified as having "any improvement" if any one of the multiple medication trials in each category resulted in any improvement in their pain.
