# Supplementary material for: Leucine‐Rich Glioma‐Inactivated 1 versus Contactin‐Associated Protein‐like 2 Antibody Neuropathic Pain: Clinical and Biological Comparisons
Source: Ann Neurol. 2021 Aug 30;90(4):683–90. doi: 10.1002/ana.26189 (PMC8581990; doi:10.1002/ana.26189)
Supplement: Supplementary file 1 — TABLE S1. Comparison of leucine‐rich glioma‐inactivated 1 (LGI1) and contactin‐associated protein‐like 2 (CASPR2) antibody–positive patients with pain. aFisher exact test was used to compare binary discrete variables between the LGI1 and CASPR2 pain groups. The Mann–Whitney test was used to compare continuous variables. Probability values are not corrected for multiple comparisons. Double‐positive patients were excluded from statistical analysis due to the small sample size. bDouleur Neuropathique 4 (DN4) scores out of 10 (2 points were discounted for lack of physical examination). Scores > 3 are considered to represent neuropathic pain in the absence of physical examination. cPatients who had multiple trials of conventional analgesia or immunotherapy agents were classified as having "any improvement" if any one of the multiple medication trials in each category resulted in any improvement in their pain. [file ANA-90-683-s001.docx]

**Supplementary Table: Comparison of LGI- and CASPR2 antibody-positive patients with pain.** ^a^Fisher’s exact test was used to compare binary discrete variables between the LGI1 and CASPR2 pain groups. The Mann-Whitney test was used to compare continuous variables. P values are not corrected for multiple comparisons. Double positive patients were excluded from statistical analysis due to the small sample size. ^b^DN4 scores out of 10 (2 points discounted for lack of physical examination). Scores over 3 considered as neuropathic pain in the absence of physical examination. ^c^Patients who had multiple trials of conventional analgesia or immunotherapy agents were classified as having ‘any improvement’ if any one of the multiple medication trials in each category resulted in any improvement in their pain.

Abbreviations: CASPR2 contactin associated protein 2; CNS central nervous system; DN4 Douleur neuropathique 4; LGI1 leucine rich glioma inactivated 1; PNH peripheral nerve hyperexcitability; PNS peripheral nervous system; PROMIS-PI patient-reported outcome measurement information system pain interference; VAS visual analogue scale

|  | LGI1-Ab^+^ | CASPR2-Ab^+^ | LGI1/CASPR2-Ab^+^ double positive | Comparison between LGI1-Ab^+^ and CASPR2-Ab^+a^  (p values) |
| --- | --- | --- | --- | --- |
| **Total cohort** | | | | |
| Number of patients | 108 | 33 | 6 | **NA** |
| Age of onset.  median; range | 64 (24-92) | 68 (19-82) | 54 (42-75) | 0.0875 |
| Gender.  Proportion (%) | 72/108 (67%) | 30/33 (91%) | 2/6 (33%) | **0.0069**** |
| **Patients with pain** | | | | |
| Total number of patients with pain/ patients phenotyped.  Proportion (%) | 20/108 (19) | 17/33 (52) | 2/6 (33) | **0.0005***** |
| Age of onset  median; range | 61; 27-92 | 68, 28-82 | 66; 56-75 | **0.038*** |
| Gender.  male/total (%) | 14/20 (70%) | 16/17 (94%) | 1/2 (50%) | 0.098 |
| HLA-DRB1*07:01 | 10/11 (91%) | 2/17 (12%) | 0/1 (0%) | **<0.0001****** |
| HLA-DRB1*11:01 | 0/11 (0%) | 9/17 (53%) | 0/1 (0%) | **0.0039**** |
| Follow up duration (months)  median; range | 66; 12-204 | 57; 24-132 | 49; 38-59 | 0.715 |
| Isolated pain.  Proportion (%) | 1/20 (5%) | 1/17 (6%) | 0/2 (0%) | >0.999 |
| Pain with concurrent PNH.  Proportion (%) | 6/20 (30%) | 8/17 (47%) | 2/2 (100%) | 0.328 |
| Concurrent PNS and CNS/ systemic manifestations at onset.  Proportion (%) | 12/20 (60%) | 14/17 (82%) | 2/2 (100%) | 0.169 |
| CNS/ systemic manifestations at onset followed by PNS.  Proportion (%) | 7/20 (35%) | 2/17 (12%) | 0/2 (0%) | 0.137 |
| Time from disease onset to onset of pain (months).  Median; range | 0; 0-84 | 0; 0-27 | 0; 0-0 | 0.1285 |
| Number of pain patients with completed questionnaires.  Proportion (%) | 12/20 (60%)  Unable to contact 6/20 (30%)  Deaths 2/20 (10%) | 9/17 (53%)  Unable to contact 8/17 (47%) | 2/2 (100%) | 0.746 |
| Pain involving limbs.  Proportion (%) | 9/12 (75%) | 7/9 (78%) | 2/2 (100%) | >0.999 |
| Pain involving trunk.  Proportion (%) | 5/12 (42%) | 0/9 (0%) | 0/2 (0%) | 0.0451 |
| Pain involving face/neck. Proportion (%) | 2/12 (17%) | 2/9 (22%) | 0/2 (0%) | >0.999 |
| DN4 score.^b^ Median; range. | 3; 0-6 | 5; 1-6 | 4; 4-4 | 0.319 |
| Number of patients classified as having neuropathic pain. Proportion (%) | Neuropathic pain: 7/12 (58%)  Unlikely neuropathic pain: 5/12 (42%) | Neuropathic pain: 8/9 (89%)  Unlikely neuropathic pain: 1/9 (11%) | Neuropathic pain: 2/2 (100%)  Unlikely neuropathic pain: 0/2 (0%) | 0.1778 |
| Number of patients prescribed conventional analgesia. Proportion (%) | 6/12 (50%) | 5/9 (56%) | 1/2 (50%) | >0.999 |
| Number of conventional analgesics trialled/ patient.  Median; range | 2; 1-4 | 2; 1-6 | 3; 3-3 | 0.987 |
| Response to conventional analgesia.  Proportion of medication trials (%)  Proportion of patients^c^ (%) | 14 medications trialled in 6 patients  NR/worsening:  7/14 (50%)  Any improvement: 7/14 (50%)  NR/worsening:  3/6 (50%)  Any improvement  3/6 (50%) | 13 medications trialled in 5 patients  NR/worsening:  7/13 (54%)  Any improvement: 6/13 (46%)  NR/worsening: 1/5 (20%)  Any improvement:  4/5 (80%) | 3 medications trialled in 1 patient  NR/worsening:  0/3 (0%)  Any improvement: 3/3 (100%)  NR/worsening:  0/1 (0%)  Any improvement  1/1 (100%) | >0.999  >0.999 |
| Number of patients with pain prescribed immunotherapy. Proportion (%) | 10/12 (83%) | 7/9 (78%) | 2/2 (100%) | >0.999 |
| Number of immunotherapeutic agents trialled/patient.  Median; range | 2; 1-4 | 2; 1-3 | 2.5; 1-4 | 0.551 |
| Response to immunotherapy medication.  Proportion of medication trials (%)  Proportion of patients^c^ (%) | 22 medications trialled in 10 patients  NR/worsening:  4/22 (18%)  Any improvement: 18/22 (82%)  NR/worsening:  1/10 (10%)  Any improvement:  9/10 (90%) | 13 medications trialled in 7 patients  NR/worsening:  8/13 (62%)  Any improvement: 5/13 (38%)  NR/worsening:  2/7 (29%)  Any improvement:  5/7 (71%) | 5 medications trialled in 2 patients  NR/worsening:  2/5 (40%)  Any improvement: 3/5 (60%)  NR/worsening:  0/2 (0%)  Any improvement  2/2 (100%) | **0.024***  0.537 |
| PROMIS pain interference scale (out of 40) at most severe.  Median; range | 26; 8-40 | 33; 8-40 | 26.5; 13-40 | 0.6621 |
| PROMIS pain interference scale (out of 40) post immunotherapy.  Median; range | 8; 8-22 | 19.5; 8-40 | 10.5; 8-13 | **0.006*** |
| PROMIS pain interference scale (out of 40) at latest follow-up.  Median; range | 8; 8-39 | 23; 8-40 | 10.5; 8-13 | **0.025*** |
| PROMIS sleep interference scale (out of 40). Median; range | 20; 8-35 | 13; 9-30 | 19; 9-29 | 0.371 |
| Did your pain contribute to your sleep issues? Proportion (%) | 4/12 (33%) | 5/9 (56%) | 0/2 (0%) | 0.396 |
| EQ5D-5L impaired mobility. Proportion (%) | No concerns:  9/12 (75%)  Any concerns:  3/12 (25%) | No concerns:  1/9 (11%)  Any concerns:  8/9 (89%) | No concerns:  2/2 (100%)  Any concerns:  0/2 (0%) | **0.008**** |
| EQ5D-5L impaired self-care. Proportion (%) | No concerns:  9/12 (75%)  Any concerns:  3/12 (25%) | No concerns:  3/9 (33%)  Any concerns:  6/9 (67%) | No concerns:  2/2 (100%)  Any concerns:  0/2 (0%) | 0.087 |
| EQ5D-5L impaired function with usual activities.  Proportion (%) | No concerns:  7/12 (58%)  Any concerns:  5/12 (42%) | No concerns:  0/9 (0%)  Any concerns:  9/9 (100%) | No concerns:  1/2 (50%)  Any concerns:  1/2 (50%) | **0.007**** |
| EQ5D-5L pain.  Proportion (%) | No concerns:  5/12 (42%)  Any concerns:  7/12 (58%) | No concerns:  1/9 (11%)  Any concerns:  8/9 (89%) | No concerns:  1/2 (50%)  Any concerns:  1/2 (50%) | 0.178 |
| EQ5D-5L anxiety/ depression.  Proportion (%) | No concerns:  6/12 (50%)  Any concerns:  6/12 (50%) | No concerns:  0/9 (0%)  Any concerns:  9/9 (100%) | No concerns:  2/2 (100%)  Any concerns:  0/2 (0%) | **0.019*** |
| EQ5D-VAS.  Median; range | 78; 20-95 | 35; 15-80 | 80; 70-90 | **0.019*** |
